# Letter from the Editor-in-Chief

**DOI:** 10.19102/icrm.2017.081202

**Published:** 2017-12-15

**Authors:** Moussa Mansour


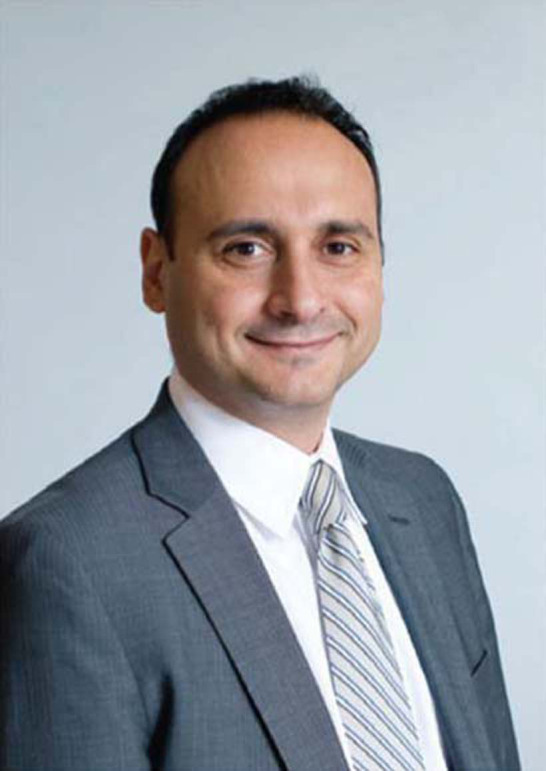


Dear Readers,

The December issue of *The Journal of Innovations in Cardiac Rhythm Management* is my most favorite issue of the year. It often contains articles reflecting on the major discoveries in the field of cardiac electrophysiology that occurred in the last year. In addition, this December’s issue contains an important original research article by Doshi et al. titled “Predictors of Elevated Defibrillation Threshold with the Subcutaneous Implantable Cardioverter-defibrillator.” The authors analyzed the defibrillation threshold (DFT) in 50 patients who underwent subcutaneous implantable cardioverter-defibrillator (S-ICD) implantation and found that increased body mass index, body surface area, and septal or posterior wall thickness were associated with high DFT.

The introduction of the S-ICD represented a major step forward in the field of cardiac defibrillation. This technology provides many advantages in comparison with conventional devices, mostly due to the associated elimination of intravascular leads. While all features of conventional ICDs have been studied extensively, data regarding some aspects of S-ICD use, including DFT, remain scarce. The study mentioned above included a small number of patients; however, it has important clinical implications and it highlights the need to develop advanced screening tools to identify patients who may have high DFT.

I would like to end this letter by thanking both the authors who submitted manuscripts and the peer reviewers who provided their thoughts on these manuscripts for inclusion in *The Journal of Innovations in Cardiac Rhythm Management* this past year. Our authors and peer reviewers are integral to the success of the journal’s efforts to ensure that only manuscripts of the highest quality that discuss relevant topics within the electrophysiology industry are published. I would also like to thank the editorial team whose dedication, skill, and professionalism have been critical for the delivery of the high level of educational content of this publication.

Best wishes for happy holidays and a healthy New Year.

Sincerely,


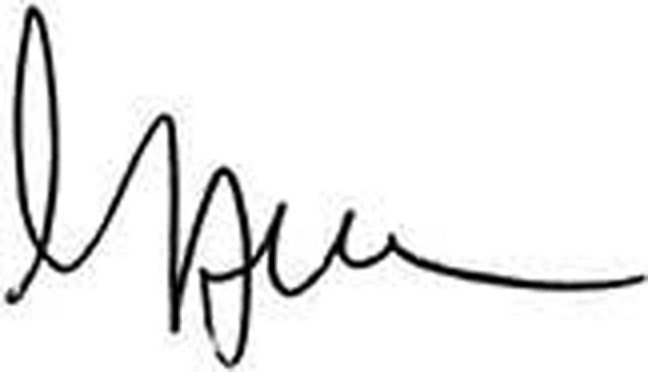


Moussa Mansour, MD, FHRS, FACC

Editor-in-Chief

The Journal of Innovations in Cardiac Rhythm Management

MMansour@InnovationsInCRM.com

Director, Atrial Fibrillation Program

Jeremy Ruskin and Dan Starks Endowed Chair in Cardiology

Massachusetts General Hospital

Boston, MA 02114

